# Role of Extracellular Vesicles in Glia-Neuron Intercellular Communication

**DOI:** 10.3389/fnmol.2022.844194

**Published:** 2022-04-13

**Authors:** Shahzad Ahmad, Rohit K. Srivastava, Pratibha Singh, Ulhas P. Naik, Amit K. Srivastava

**Affiliations:** ^1^Department of Medical Elementology and Toxicology, Jamia Hamdard University, New Delhi, India; ^2^Department of Pediatric Surgery, Texas Children’s Hospital, Houston, TX, United States; ^3^M.E. DeBakey Department of Surgery, Baylor College of Medicine, Houston, TX, United States; ^4^Department of Biochemistry and Cell Biology, Biosciences Research Collaborative, Rice University, Houston, TX, United States; ^5^Department of Medicine, Sidney Kimmel Medical College, Thomas Jefferson University, Cardeza Foundation for Hematologic Research, Philadelphia, PA, United States

**Keywords:** extracellular vesicles, glia, neuron, intercellular communication, cellular cargo

## Abstract

Cross talk between glia and neurons is crucial for a variety of biological functions, ranging from nervous system development, axonal conduction, synaptic transmission, neural circuit maturation, to homeostasis maintenance. Extracellular vesicles (EVs), which were initially described as cellular debris and were devoid of biological function, are now recognized as key components in cell-cell communication and play a critical role in glia-neuron communication. EVs transport the proteins, lipids, and nucleic acid cargo in intercellular communication, which alters target cells structurally and functionally. A better understanding of the roles of EVs in glia-neuron communication, both in physiological and pathological conditions, can aid in the discovery of novel therapeutic targets and the development of new biomarkers. This review aims to demonstrate that different types of glia and neuronal cells secrete various types of EVs, resulting in specific functions in intercellular communications.

## Introduction

Two-way intercellular communication between glia and neurons is essential for the optimal functioning of the central nervous system (CNS) (Fields and Stevens-Graham, 2002; Basso and Bonetto, 2016). The intercellular communication between glia and neurons is bidirectional and is mediated through ion fluxes, neurotransmitters, cell adhesion molecules, and secretomes (Pascual et al., 2020). Extracellular vesicles (EVs) have emerged as vital intermediaries for glia-neuron communication and are among the most important constituents of secretomes (Koniusz et al., 2016; Lizarraga-Valderrama and Sheridan, 2021).

**FIGURE 1:**
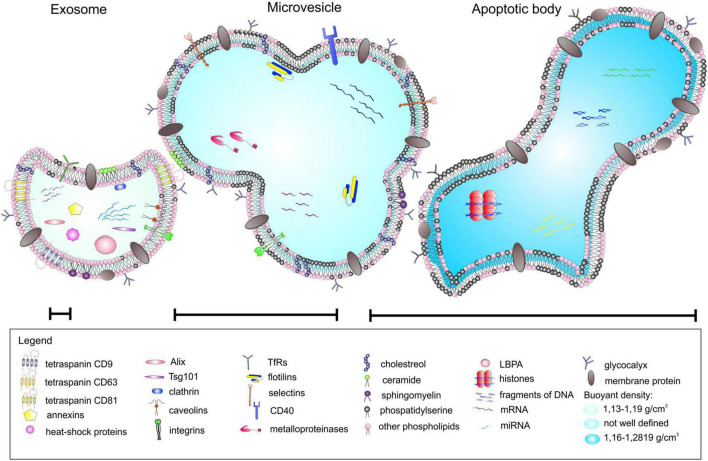
Different types of extracellular vesicles (EVs). EVs can be categorized into three main classes based on their mode of origin: (i) exosomes, (ii) microvesicles, and (iii) apoptotic bodies. An EV cargo consists of a specific set of proteins, lipids, and nucleic acids, and neighboring cells use EVs as a method of paracrine transfer of molecular signals between cells. Figure reproduced from Koniusz et al. (2016).

Extracellular vesicles are membrane-surrounded structures released by most cell types and are characterized by a specific set of proteins, lipids, and nucleic acids (Koniusz et al., 2016; Harting et al., 2018; Lopez et al., 2019). They are broadly categorized based on their biological function and biogenesis into exosomes (30–120 nm), microvesicles (100–1000 nm), and apoptotic bodies (50–4000 nm) (Figure 1). The last two represent the heterogeneous populations of vesicles generated by the external budding of the plasma membrane. Exosomes, in contrast, are generated as intraluminal vesicles through the inward budding of the multivesicular bodies (Stoorvogel et al., 2002; Gould and Raposo, 2013; Koniusz et al., 2016). Historically, EVs have been defined as membrane debris that shuttle cellular waste from various cell types into the extracellular space with no real biological significance (Graykowski et al., 2020). Over time, studies have shown that EVs can stimulate adaptive immune responses, and subsequent studies have identified the importance of EVs in intracellular communication. Both glia and neurons release EVs that contain cargos such as proteins, nucleic acids, and lipid signaling molecules (Agnati et al., 2010; Guo et al., 2010; Lippi et al., 2016). These EV cargos may have potential roles in transcriptional and translational regulation with putative influence on downstream signaling pathways in recipient cells (Ung et al., 2014). Therefore, EV-mediated intercellular communication between glia and neuron likely results in changes in the transcriptome and proteome of target cells and serves as an important method of information transfer between them. In this review, we highlight and discuss the recent studies of EV-mediated glia-neuron communication in the CNS.

## Glia-Neuron Intercellular Communication

**FIGURE 2:**
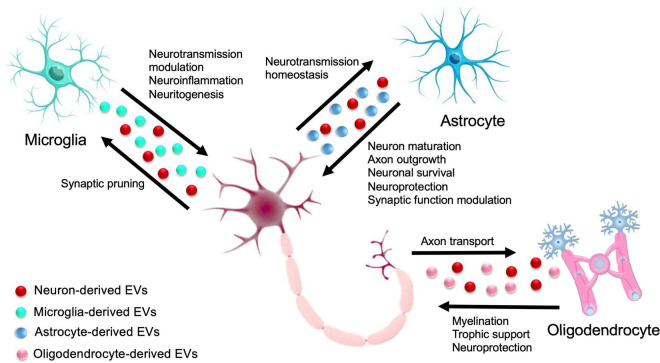
Extracellular Vesicle-mediated glia-neuron intercellular communication in the central nervous system (CNS). In the CNS, cross talk between glia and neurons is crucial for a variety of biological functions, ranging from neuroprotection, neural circuit maturation, homeostasis maintenance, and synaptic function modulation. Solid arrows indicate the exchange of EVs between different glial cells and neurons.

The functions of the CNS, including synaptic transmission, axonal conduction, and information processing, depend on the two-way communication between neurons and glial cells. Glial cells regulate various functions of neurons, such as synapse formation, the strengthening of synapses, and information processing. Conversely, neurons regulate different glia activities, including proliferation, differentiation, and myelination of glial cells (Fields and Stevens-Graham, 2002). This interdependence of neuron and glial activities suggests a systemic mechanism of bidirectional glia-neuron communication (Figure 2).

Glial cells are the non-neuronal cells in the CNS and the peripheral nervous system (PNS). They do not produce electrical impulses, but they are the active participants in CNS physiology (Pontén, 1975; Travis, 1994; Fields et al., 2015). In the CNS, glial cells include astrocytes, microglia, NG2 glia, oligodendrocytes, radial glial cells (RGCs), and ependymal cells. In addition, pituicytes from the posterior pituitary share common characteristics with astrocytes, and tanycytes are special ependymal cells in the median eminence of the hypothalamus. In the PNS, glial cells include Schwann cells (SCs), satellite glial cells (SGCs), and enteric glial cells (Jessen, 2004; Clasadonte and Prevot, 2018; Rodríguez et al., 2019).

In the CNS, astrocytes exhibit heterogeneous functions and morphology depending on their location (Khakh and Sofroniew, 2015; Ben Haim and Rowitch, 2017). They also participate in neuronal functions, including supplying energy metabolites and maintaining water ion homeostasis. Astrocytes are actively involved in calcium excitability, tripartite synapse, neurovascular coupling, and maintenance of the blood-brain barrier (BBB) (Filosa et al., 2016). Microglia are prime immune cells of myeloid origin. They also contribute to synaptic pruning during development and synaptic modulation. These glial cells have immunocompetent potential and are known as phagocytic cells of the CNS (Prinz et al., 2019). Microglia originate from yolk sac progenitors and contribute to synaptic pruning during development and synaptic modulation (Wu et al., 2015; Hong and Stevens, 2016). NG2 glial cells, also known as oligodendrocytes progenitor cells (OPCs) (Ffrench-Constant et al., 1986; Dimou and Götz, 2014), first appear in the early phases of development and are present in the adult CNS (Kirdajova and Anderova, 2020). OPCs are the precursors of oligodendrocytes that produce myelin to insulate axons, enable fast salutatory impulse propagation, and provide metabolic support to myelinated axons (Saab et al., 2013; Moore et al., 2020). Radial cells have a periventricular cellular body and extend an elongated process, directing the newly formed neurons to their destinations (Kriegstein and Alvarez-Buylla, 2009; Sharif et al., 2018). With the various physiological functions of neuroendocrine, neurogenic, and metabolic processes, tanycytes are specialized ependymoglial cells derived from radial cells. Tanycytes are found in the third ventricle and on the floor of the fourth ventricle and have processes extending into the hypothalamus (Wittkowski, 1998; Barry et al., 2014). These glial cells are crucial in determining the functional interactions of specific neuronal subpopulations involved in the control of metabolism (García-Cáceres et al., 2019). Pituicytes are the dominant, non-neuronal elements found in the posterior lobe of the pituitary. Pituicytes are astrocyte-like cells that enclose neurosecretory fiber terminals *via* their processes, remodel glia-neurons to regulate the access of magnocellular neurosecretory axons directly to the pericapillary space, and control the potential paracrine and autocrine actions of secreted peptides (Hatton et al., 1984; Wittkowski, 1998; Theodosis et al., 2008).

In the PNS, glial cells perform a plethora of functions linked to axon regeneration, myelination, neuronal support, regulation of synaptic connectivity, and sensory function (Kastriti and Adameyko, 2017; López-Leal et al., 2020). Current evidence shows that SCs, the main glial cell in the PNS, possess a remarkable regenerative potential, and, in addition to their roles in myelination, SCs play a critical role in energetic/metabolic support of axons to maintain their integrity and function (Beirowski et al., 2014; Salzer, 2015; Wong et al., 2017; Babetto et al., 2020).

The above outline of diverse functions and types of glial cells demonstrates a synchronized mechanism of glia-neuron communication. Recent literature has identified the key role of EVs in glia-neuron communication. EVs have the potential to modify the morphology and function of target cells upon delivery of the messages. However, more research needs to be done to establish the precise role of EVs in glia-neuron communication.

## Historical Background of Extracellular Vesicles in Glia-Neuron Communication

Although extracellular and vesicular roles of EVs were recognized during the 1940s–1950s, Bonucci (1970) was the first to coin the term “extracellular vesicles” (Chargaff and West, 1946; Anderson, 1969; Bonucci, 1970; Yáñez-Mó et al., 2015). In 1970, Grillo’s proposal of the role of merocrine and apocrine secretory processes in neuronal signaling was criticized due to its experimental nature (Grillo, 1970; Dermietzel et al., 1972). In 1996, a complex signaling function of EVs was established. Later on, several studies in the first decade of the 21st century reported the intercellular transfer of nucleic acid through EVs.

Almost all types of cells in the body naturally release some lipid bilayer-delimited particles called EVs, which vary in function and chemical composition (Théry et al., 2018). Aside from the transfer of receptors, bioactive lipids, proteins, and nucleic acid, EVs also play a key role in the regulation of homeostasis and immune functions (Koniusz et al., 2016) and the transfer of biomolecules in glia-neuron intercellular communication. Moreover, functionally transferred RNA in recipient cells was reported (Baj-Krzyworzeka et al., 2006; Ratajczak et al., 2006; Aliotta et al., 2007; Valadi et al., 2007; Skog et al., 2008; Pegtel et al., 2010; Yáñez-Mó et al., 2015). The significant impacts of EVs in communication within the CNS include *trans*synaptic and interneuronal communication (Budnik et al., 2016; Veziroglu and Mias, 2020). Astrocytes have been shown to cause neuronal apoptosis in *in vitro* studies (Söllvander et al., 2016; Nikitidou et al., 2017). Additionally, EVs carrying cargo have been associated with neuronal growth and survival, synaptic transmission, regulation, and neurodegeneration (Agnati and Fuxe, 2014).

## Various Kinds of Extracellular Vesicles in Glia-Neuron Intracellular Communication

The deep involvement of EVs in CNS physiology has been increasingly demonstrated in recent literature. EVs in CNS research have opened new perspectives and emerged as crucial players in neuron-glial communication, regulating the circulation of pathogenic factors, inflammation, cargo transport, neurotransmission, axonal integrity, and support neurons (López-Guerrero et al., 2020). EVs are derived from all kinds of CNS cells, including neurons and non-neuronal glial cells, astrocytes, microglia, oligodendrocytes, NG2 cells, RGCs, tanycytes, and pituicytes. EVs derived by these cells transfer the proteins, lipids, and nucleic acid cargo, participate in neuron-glia communication, and relay even more complex messages. Table 1 demonstrates the role of EVs derived from different types of CNS cells. The role of EVs derived from different neurons and glial cells in glia-neuron communications is summarized next.

**TABLE 1:** Role of extracellular vesicles (EVs) derived from different types of central nervous system (CNS) cells in glia-neuron intercellular communication.

S. No.	Type of cell secreting extracellular vesicles	Functions	References
1.	Neuron	*Trans* synaptic communication, removal of microglia of degenerating neuritis	Bahrini et al., 2015; Graykowski et al., 2020
2.	Astrocytes	Neuron maturation and survival, and modulation of synaptic function, transport mtDNA and miRNA, ATP, Hsp/Hsc70 and synapsin I, neuro-protection, reduced neuronal cell death, regulation of autophagy, brain damage repair, neurons morphology, dendritic development and synaptic homeostasis, regulation of signaling of gap junction and CREB, transport of neurotoxic factors, loss of excitatory and inhibitory synapses	Proia et al., 2008; Guescini et al., 2010; Pegtel et al., 2010; Frühbeis et al., 2013b; Agnati and Fuxe, 2014; Jovičić and Gitler, 2017; Saribas et al., 2017; Chaudhuri et al., 2018; Durkee and Araque, 2019; Katuri et al., 2019; Xu et al., 2019; Chen et al., 2020; Datta Chaudhuri et al., 2020; Hu et al., 2020; Luarte et al., 2020
3.	Oligodendrocytes	Pathological functions, bidirectional neuron-glia communication, transport of proteolipid protein, 2′,3′-Cyclic nucleotide 3′-phosphodiesterase (CNP), myelin basic protein, and myelin-oligodendrocyte glycoprotein, metabolites, protective proteins, glycolytic enzymes, mRNA, and miRNA, axonal integrity, neuro-protection, promotion of fast axonal transport and its maintenance in starving neurons	Krämer-Albers et al., 2007; Falchi et al., 2013; Frühbeis et al., 2013a; Fröhlich et al., 2014; Frühbeis et al., 2020; Krämer-Albers, 2021
4.	Microglia	Neurodegenerative processes, detrimental and protective role in myelin injuries, enhancement in excitatory transmission, neuronal production and modulation of synaptic activity, neuro-inflammation, transport of endocannabinoid N-arachidonoylethanolamine, aminopeptidase CD13 and the lactate transporter monocarboxylate transporter-1 (MCT-1) markers, neurodegeneration in amyotrophic lateral sclerosis, TNF production	Potolicchio et al., 2005; Antonucci et al., 2012; Turola et al., 2012; Gabrielli et al., 2015; Paolicelli et al., 2019; Christoforidou et al., 2020; Pascual et al., 2020; Raffaele et al., 2020

### Neuron-Derived Extracellular Vesicles

Early evidence of the release of EVs from neurons was demonstrated using primary cell cultures of cortical neurons in rat and mice embryos (Fauré et al., 2006). EVs derived from neurons at the synapses may be taken up by other neurons, suggesting the involvement of EVs in *trans*synaptic communication. Cortical neurons in the mammalian nervous system have been reported to release EVs triggered by increased postsynaptic calcium levels due to synaptic glutamatergic activity (Graykowski et al., 2020). Potassium-induced depolarization has been reported to enhance the release of EVs from neurons contributing to the removal of microglia from degenerating neurites by the upregulation of microglial complement molecule C3 (Bahrini et al., 2015). Recent studies show that neuron-derived EVs carry mRNA and proteins, which further proves the role of EVs in synaptic communication (Ashley et al., 2018). Presynaptic release of EVs is also shown to modulate retrograde signaling by the postsynaptic cell, which may be important during CNS development, axon guidance, or synaptic plasticity (Korkut et al., 2013).

### Glia-Derived Extracellular Vesicles

Glial cells are distributed throughout the CNS and comprise various populations of cells with different origins, functions, and structures. Recent studies on glial cells derived from EVs suggest that EVs are a key player in intercellular communication and CNS function and dysfunction.

#### Astrocytes-Derived Extracellular Vesicles

Astrocytes are the most abundant type of glial cells, and studies have reported that astrocyte-derived EVs (ADEVs) are key players in glia-neuron communication. They have been found to contribute to neuron maturation and the survival and modulation of synaptic function (Durkee and Araque, 2019). ADEVs vary in size ranging from 150 to 500 nm and carry various transfer molecules, including ATP, Hsp/Hsc70, and synapsin I and angiogenesis modulating factors, such as fibroblast growth factor 2 (FGF2), vascular endothelial growth factor (VEGF), pigment epithelium-derived factor (PEDF), and endostatin (Proia et al., 2008; Pegtel et al., 2010; Frühbeis et al., 2013b; Agnati and Fuxe, 2014). Studies have shown that ADEVs also transport mitochondrial DNA (Guescini et al., 2010) and microRNA (miRNA), which contribute to intercellular communication (Jovičić and Gitler, 2017). The neuroprotecting properties of ADEVs have been shown to be effective against hypoxia, ischemia, oxidative stress, and hypoglycemia through prion protein-dependent mechanisms (Guitart et al., 2016; Pascua-Maestro et al., 2019). Reduced neuronal cell death has been reported through the exposure of ADEVs to oxygen and glucose deprivation (Xu et al., 2019). Regulation of autophagy by ADEVs has demonstrated apoptosis inhibition in neurons (Pei et al., 2019). Moreover, the role of ADEVs in traumatic brain injuries was recently shown to contribute to neuroprotection and damage repair through mitochondrial function, restoration, and apoptosis downregulation (Chen et al., 2020).

Neuronal morphology, dendritic development, and synaptic homeostasis have been proven to be directly regulated by ADEVs through modification of their miRNA cargo (Chaudhuri et al., 2018; Luarte et al., 2020). It also appears that ADEVs influence neurite outgrowth, guide axons, are involved in the potentiation, and help in the biogenesis of synapses. Furthermore, the treatment of ADEVs with interleukin-10 (IL-10) regulates the signaling of gap junction and cAMP-response element binding protein (CREB) (Datta Chaudhuri et al., 2020). Along with the different proteins, lipids, and nucleotide cargo, ADEVs also carry neurotoxic factors such as human immunodeficiency virus (HIV)-related neurotoxic proteins (Katuri et al., 2019).

Various proteins have been reported to damage the CNS. One example is negative regulatory factor (Nef) protein. Nef is a small protein expressed abundantly in astrocytes of HIV-1-infected brains and are released in ADEVs. These Nef-containing ADEVs play a significant role in the pathogenesis of HIV-associated neurological disorders (Saribas et al., 2017).

Additionally, the loss of excitatory and inhibitory synapses can be due to an uptake of ADEVs by hippocampal neurons through increased expression and the release of several miRNAs (Hu et al., 2020). A recent study also observed the modulation of neuronal uptake, differentiation, and firing by activated human ADEVs (You et al., 2019). Current literature has demonstrated the therapeutic and toxic potential of ADEVs, which suggests the need for further investigation on the impacts of ADEVs on glia-neuron communication.

#### Oligodendrocyte-Derived Extracellular Vesicles

Multifunctional oligodendrocytes insulate the axon by producing the myelin sheath, which depends on bidirectional glia-neuron communication (Sherman and Brophy, 2005; Nave, 2010). Other functions of oligodendrocytes include controlling the extracellular ion balance, participating in the BBB, participating in the repairing and scarring processes after CNS injuries, and providing trophic support to neurons. Oligodendrocytes release EVs and heterogeneous compositions of oligodendrocyte-derived EVs (ODEVs), which have been proven to play a crucial role in pathological functions (Falchi et al., 2013). A novel mode of bidirectional glia-neuron communication through ODEVs, particularly exosomes, has been established in a mouse model (Frühbeis et al., 2013a). ATP-triggered activation of P2 × 7 receptors and the subsequent action of acid sphingomyelinase evoke ODEV secretion, particularly in microvesicles (Bianco et al., 2009). Calcium treatment induces the release of ODEVs and is reported to carry major proteins involved in myelin, such as proteolipid protein, CNP, myelin basic protein, and myelin-oligodendrocyte glycoprotein (Krämer-Albers et al., 2007). Interestingly, the ODEVs lacking these proteins have been suggested to be impaired (Frühbeis et al., 2013a). Studies show that the promyelinating effect of the neuronal-conditioned medium is counteracted by ODEVs (Reiter and Bongarzone, 2020). The release of glutamate by electrically active axons stimulates the calcium ion entry through oligodendroglial glutamate receptors, which activates the release of ODEVs, particularly exosomes and neurons, and internalizes them by using their cargo (Frühbeis et al., 2013a). Active neurons pass demand signals to oligodendrocytes to deliver the supportive biomolecules through ODEVs, which transfer metabolites, protective proteins, glycolytic enzymes, mRNA, and miRNA to axons contributing to the maintenance of axonal integrity (Frühbeis et al., 2013a). The uptake of ODEVs by neurons results in EV-content retrieval, leading to multiple effects on neurons. Subsequently, the therapeutic effects of ODEVs on stressed neurons include restored, faster axonal transport compared to untreated neurons and neurons treated with EVs from other sources. Therefore, studies suggest that the neuroprotective effects of ODEVs maintain vital cellular functions (Frühbeis et al., 2013a; Fröhlich et al., 2014; Krämer-Albers, 2021). The promotion of fast axonal transport and its maintenance in starving neurons have also been associated with ODEVs (Frühbeis et al., 2020). Together, these studies indicate that ODEVs contribute a wide range of functions in glia-neuron communication, including long-term maintenance of neurons, axonal transport, myelin diseases, and loss of axonal integrity.

#### Microglia-Derived Extracellular Vesicles

Microglia are found throughout the brain and the spinal cord and act as the first line of defense in the brain as resident macrophages (Lawson et al., 1992; Kreutzberg, 1995; Ginhoux et al., 2013; Filiano et al., 2015). Studies report that intercellular communication by microglia in both physiological and pathological conditions involves biomolecules secreted through microglial EVs (MGEVs) (Paolicelli et al., 2019). Several studies report the existence of MGEVs (Verderio et al., 2012; Garzetti et al., 2014) and their active role in glia-neuron communication in various pathological and physiological conditions, including neurodegenerative processes (Paolicelli et al., 2019). Recent studies report the detrimental and protective effects of MGEVs on myelin injuries. Enhanced excitatory transmission, neuronal production, and modulation of synaptic activity through ceramide and sphingosine synthesis induction have been associated with the interaction of MGEVs and neurons, which suggests the role of MGEVs in glia-neuron communication (Antonucci et al., 2012; Turola et al., 2012; Paolicelli et al., 2019). MGEVs, particularly exosomes, have been associated with various mental disorders, such as depression, anxiety, bipolar disorder, and schizophrenia (Saeedi et al., 2019). Recent studies suggested the neuroinflammatory role of misfolded and inflammatory proteins and the neurotoxic potential of MGEVs (Pascual et al., 2020). Additionally, the endocannabinoid *N*-arachidonoylethanolamine are carried by MGEVs, stimulating the cannabinoid receptor 1 of GABAergic neurons and contributing to the inhibition of presynaptic transmissions (Gabrielli et al., 2015). Proteomic analysis suggests that MGEVs carry some unique markers, such as aminopeptidase CD13 and the lactate transporter MCT-1 (Potolicchio et al., 2005). Regulation and propagation of neuroinflammatory responses in the CNS have also been associated with MGEVs through pro-inflammatory cytokines and glyceraldehyde-3-phosphate dehydrogenase (GAPDH) (Bianco et al., 2005; Takenouchi et al., 2015). Subsequently, the treatment of microglia with lipopolysaccharide has also been associated with altered production of MGEVs (Yang et al., 2018). Additionally, MGEVs with P2 × 7 receptors reduce apoptosis (Turola et al., 2012), which suggests the need to study MGEVs for potential biomarkers of various chronic neurodegenerative diseases (Panaro et al., 2020; Aires et al., 2021). In Alzheimer’s disease, MGEVs have positive and negative effects (Trotta et al., 2018). Similarly, miRNA carried by MGEVs is associated with neurodegeneration in amyotrophic lateral sclerosis (Christoforidou et al., 2020). Tumor necrosis factor production through MGEVs was also found to alter neuronal functions (Raffaele et al., 2020). Recent studies also associated the communication of neural progenitor cells (NPCs) and microglia with MGEVs and EVs released from NPCs (Cossetti et al., 2012; Pluchino and Cossetti, 2013; Cossetti et al., 2014; Matarredona et al., 2018).

#### NG2 Glial Cells-Derived Extracellular Vesicles

The majority of proliferative cells outside neurogenic niches in the adult CNS comprise neural progenitors expressing chondroitin sulfate proteoglycan 4, which are known as NG2 glial cells (also referred to as OPCs or polydendrocytes) (Nishiyama et al., 1999; Dawson et al., 2003; Nishiyama et al., 2014; Nakano et al., 2017). NG2 glial cells have an important role in remyelination as they have the ability to proliferate and differentiate after a demyelinating insult. In CNS injuries and neurodegenerative diseases, NG2 glial cells rapidly proliferate and migrate to restore their population in focal cellular loss (McTigue et al., 2001; Nishiyama et al., 2009; Kang et al., 2010; Hughes et al., 2013). NG2-glial cells-derived EVs are reported as the important intercellular transporter of retinoic acid and enable the cross talk between NG2 glial cells and neurons to mediate remyelination and axonal/neurite outgrowth (Goncalves et al., 2019; Bahram Sangani et al., 2021). However, limited literature is available describing the role of EVs in neuron-NG2 glial cell communication.

#### Schwann Cells-Derived Extracellular Vesicles

Schwann cells support maintenance and regenerative responses of axons by diverse mechanisms of intercellular communication. A crucial role of SCs has been established in the regulation of a variety of passive axonal functions, including myelin formation with subsequent elevation in the conduction velocity, and active axonal functions, including sodium channel enrichment, internodal distance specifications, and metabolic maintenance (Court et al., 2004; Hartline and Colman, 2007; Nave and Trapp, 2008; Voas et al., 2009; Feldman et al., 2017).

Schwann cells-derived EVs (SDEVs) are also secreted from different phenotypic SCs and carry distinct protein and nucleic acid cargoes that exert either a neuroprotective or a pathological effect on the recipient cells (Wong et al., 2022). In addition to the classical mechanism of axonal communication of SCs, SDEVs mediate the lateral molecular cargo transfer from SCs to axons (Court et al., 2008; Lopez-Verrilli et al., 2013; López-Leal et al., 2020). SDEVs have also been found to be taken by peripheral axons to increase neurite sprouting of sensory neurons *in vitro* and to regenerate axons by 50% following neuronal injury *in vivo* (Lopez-Verrilli et al., 2013; López-Leal et al., 2020). SDEVs have also been found to carry and transfer p53 to axons (Lopez-Verrilli et al., 2013). The p75 neurotrophin receptor (p75^NTR^) plays a key role in the SC-axon myelination during development and may arbitrate cell survival and cytoskeletal remodeling *via* p53 or induce cell death *via* c-Jun N-terminal kinase pathway activation (Cosgaya et al., 2002; Chao, 2003). SDEVs play a regulatory role in the homeostasis of different cell types of the PNS through p75^NTR^ and sortilin is identified by a small RNA profile (Gonçalves et al., 2020). EVs from skin precursor-derived SCs have been demonstrated to play a crucial role in axonal regrowth and regeneration of neurons (Wu et al., 2020) and repair of peripheral nerve defects by nerve grafts (Yu et al., 2021). Similarly, the transfer of RNA through EVs secreted by SC-like differentiated adipose stem cells has been shown to promote neurite outgrowth (Ching et al., 2018). The proliferation and maintenance of human dental pulp cells have also been shown to be promoted by SDEVs (Li et al., 2022).

In addition to their physiological roles, SDEVs have also been shown to exhibit pathological impact. SDEVs from high glucose-stimulated SCs have been reported to involve in mediating the development of diabetic peripheral neuropathy in a diabetic mouse model (Jia et al., 2018). SDEVs are also reported to be involved in the development of age-related schwannomatosis (a rare genetic disorder that results in tumors that grow on the peripheral nerves throughout the body) (Chignon-Sicard et al., 2019). These compelling findings elucidate the importance of SDEVs in glia-neuron cross talk both in health and in pathological conditions.

#### Satellite Cells-Derived Extracellular Vesicles

The surface of neurons cell bodies in the ganglia of the PNS is covered by SGCs that are found in sensory, sympathetic, and parasympathetic ganglia. SGCs have a variety of functions, including the regulation of the microenvironment of sympathetic ganglia (Hanani and Spray, 2020). A recent study reported that SGCs shed vesicles in the size range of exosomes and alter their protein profile under inflammatory conditions *in vitro* (Vinterhøj et al., 2019). However, the relevance of SGC-derived EVs in glia-neuron communication is still in its infancy and requires further studies.

#### Radial Glial Cells, Tanycytes, and Pituicytes-Derived Extracellular Vesicles

Radial glial cells are progenitor cells of a bipolar shape with the specialized function of producing neurons and certain glia, including astrocytes and oligodendrocytes (Noctor et al., 2001; Rakic, 2009; Beattie and Hippenmeyer, 2017). Long radial processes of RGCs facilitate the transfer of newly produced neurons to their final destination (Rakic, 1972; Campbell and Götz, 2002). The third ventricle of the brain and the floor of the fourth ventricle possess special ependymal cells called tanycytes, which extend into the hypothalamus (Jansen et al., 1982; Wittkowski, 1998; Rodríguez-Rodríguez et al., 2019). The posterior part of the pituitary gland contains some glial cells, called pituicytes, which release and store neurohypophysial hormones (Hatton et al., 1984; Hatton, 1988).

So far, no studies have shown the role of EVs secreted from these glial cells. However, given their diverse physiological functions such as neurogenesis, neuronal migration, and maintenance of permeable neurovascular interfaces (Goodman and Hajihosseini, 2015; Anbalagan et al., 2018; Berg et al., 2018), it is imperative to explore the role of EVs secreted by these cells in future studies.

## Conclusion

In the last two decades, cell-to-cell communication through secreted EVs has been established. Similarly, new biological functions continue to be defined, emphasizing the importance of the functional cargoes transferred from one cell to another in physiological and pathological processes. In CNS disorders, intercellular communication is essential to protect neurons. The CNS entails a complex chain of events requiring coordinated short- and long-distance communication between numerous cell types, especially glial cells, in order to maintain their neuronal circuits. Unlike neurons, glial cells are electrically inexcitable. However, despite being unable to generate action potentials, glia are, in fact, highly active cells, communicating primarily through EV-mediated signals. Several studies have shown that both glia and neuronal cells release EVs and maintain communication that have an effect on the overall regulation of neurological activities. Furthermore, future studies to understand how glia operate as a system and how they interact with neural networks and subcellular domains of neurons would offer a promising strategy to gain pathogenic information and identify therapeutic targets and biomarkers for neurological disorders.

## Author Contributions

SA: literature review, manuscript writing, preparation of figures, and final approval of manuscript. RS, PS, and UN: manuscript writing and final approval of manuscript. AS: conception and design, financial support, administrative support, manuscript writing, and final approval of manuscript. All authors contributed to the article and approved the submitted version.

## Conflict of Interest

The authors declare that the research was conducted in the absence of any commercial or financial relationships that could be construed as a potential conflict of interest.

## Publisher’s Note

All claims expressed in this article are solely those of the authors and do not necessarily represent those of their affiliated organizations, or those of the publisher, the editors and the reviewers. Any product that may be evaluated in this article, or claim that may be made by its manufacturer, is not guaranteed or endorsed by the publisher.
